# Community-based directly observed therapy is effective and results in better treatment outcomes for patients with multi-drug resistant tuberculosis in Uganda

**DOI:** 10.1186/s12913-023-10120-7

**Published:** 2023-11-13

**Authors:** Rita Makabayi-Mugabe, Joseph Musaazi, Stella Zawedde-Muyanja, Enock Kizito, Katherine Fatta, Hellen Namwanje-Kaweesi, Stavia Turyahabwe, Abel Nkolo

**Affiliations:** 1 Infectious Diseases Institute, College of Health Sciences, Makerere University, P.0.Box 22418, Kampala, Uganda; 2 USAID-Defeat TB Project, University Research Co. LLC., Kampala, Uganda; 3 University Research Co. LLC., Maryland, USA; 4 Ministry of Health, National Tuberculosis, and Leprosy Program, Kampala, Uganda

**Keywords:** Community-based care, Health facility-based care, Directly observed therapy, Treatment outcomes, Drug-resistant TB, Uganda

## Abstract

**Background:**

Health facility-based directly observed therapy (HF DOT) is the main strategy for the management of patients with drug-resistant tuberculosis (DR TB) in Uganda, however, this still yields sub-optimal treatment outcomes. We set out to assess the effectiveness of community-based directly observed therapy (CB DOT) for the treatment of DR TB in Uganda.

**Methods:**

Using a previously developed patient-centered model for CB DOT, we assigned community health workers (CHWs) as primary caregivers to patients diagnosed with DR TB. CHWs administered daily DOT to patients in their homes. Once a month, patients received travel vouchers to attend clinic visits for treatment monitoring. We assessed the effectiveness of this model using a quasi-experimental pre and post-study. From December 2020 to March 2022, we enrolled adult DR-TB patients on the CB DOT model. We collected retrospective data from patients who had received care using the HF DOT model during the year before the study started. The adjusted effect of CB DOT versus HF DOT on DR TB treatment success was estimated using modified Poisson regression model with robust cluster variance estimator.

**Results:**

We analyzed data from 264 DR TB patients (152 HF DOT, 112 CB DOT). The majority were males (67.8%) with a median age of 36 years (IQR 29 to 44 years). Baseline characteristics were similar across the comparison groups, except for educational level, regimen type, and organizational unit with age being borderline. The treatment success rate in the CB DOT group was 12% higher than that in the HF DOT (adjusted prevalence ratio (aPR)= 1.12 [95%CI 1.01, 1.24], *P*-value=0.03). Males were less likely to achieve treatment success compared to their female counterparts (aPR=0.87 [95% CI 0.78, 0.98], *P*-value=0.02). A total of 126 (47.7%) of 264 patients reported at least one adverse event. The HF DOT group had a higher proportion of patients with at least one adverse event compared to the CB DOT group (90/152 [59.2%] versus 36/112 [32.1], *P*-value<0.01). The model was acceptable among patients (93.6%) and health workers (94.1%).

**Conclusions:**

CB DOT for DR-TB care is effective and results in better treatment outcomes than HF DOT. The cost-effectiveness of this model of care should be further evaluated.

**Supplementary Information:**

The online version contains supplementary material available at 10.1186/s12913-023-10120-7.

## Background

Drug-resistant tuberculosis (DR TB) continues to be a disease of public health interest globally. In 2018, there were approximately half a million new cases of DR TB reported, 78% of which were resistant to two major first-line anti-TB medicines, isoniazid and rifampicin [1]. Of these, an estimated 3.4% were new cases of TB, while 18% were previously treated TB cases. In the same year, there were about 200,000 deaths from DR TB [1]. Before the COVID-19 pandemic disrupted TB case detection and notification efforts, there was a slight increase in cases of DR TB as a proportion of all notified TB cases [1, 2].

Results from a national DR TB survey conducted in Uganda in 2010, showed an MDR TB prevalence of 1.4% and 12.1% among new and retreatment cases respectively [3]. In 2018, the World Health Organization (WHO) estimated 1,100 DR TB cases among notified pulmonary TB cases in Uganda [1]. In the same year, about 500 DR TB cases were started on treatment resulting in a 70% treatment success rate, a 15% mortality rate, and a 14% loss to follow-up rate for the 2016 treatment cohort [4], far below the national TB strategic plan (NSP) targets of 85% for treatment success and <5% for all unfavorable outcomes [5].

Uganda implements a mixed model of care for persons diagnosed with DR-TB. Unless there is an indication for hospitalization e.g. critical illness, serious adverse events, respiratory insufficiency, or need for intensified adherence support, care is ambulatory [6]. Treatment is initiated at tertiary referral hospitals, followed by daily directly observed therapy (DOT) at health facilities closest to the patient's home. Patients then return to the tertiary hospitals once every month for treatment monitoring and/or adverse event management. Patients who receive care through health-facility DOT (HF DOT) experience various inconveniences (e.g. travel and waiting times) and incur significant direct (e.g. transport costs) and indirect costs (e.g. time lost from work) that contribute to the high rates of suboptimal treatment outcomes associated with HF DOT [7, 8]. These suboptimal outcomes are a potential risk for the development and spread of further resistance to TB treatment.

In 2019, the WHO released updated guidelines for the management of DR TB that recommended the use of shorter all-oral regimens constructed using novel and repurposed drugs e.g. Bedaquiline (BDQ) and Linezolid (Lzd) respectively for all eligible patients, making community-based DOT (CB DOT) and the use of lay providers possible [9]. In response to the new recommendations from the WHO, Uganda introduced new injection-free, DR-TB treatment regimens in October 2020. These regimens included; 1) a bedaquiline-based injection-free shorter treatment regimen (the modified shorter treatment regimen) 2) individualized longer treatment regimens based on age, fluoroquinolone resistance, and site of the disease and 3) salvage regimens for patients with extensive resistance patterns [9]. The introduction of these new regimens made CB DOT, which has been documented to result in better treatment outcomes in many settings [10–13] a possibility for Uganda. We set out to assess the effectiveness of CB DOT compared to HF DOT for the management of DR-TB in Uganda.

## Methods

### Study design

In 2019, we developed a CB DOT model for DR TB based on patient preferences elicited during a previous study. In the CB DOT model, community health workers were assigned as primary caregivers and received travel vouchers to administer DOTs in patients' homes [14]. To assess the effectiveness of this model, we designed a quasi-experimental pre and post-study. It comprised of the observational prospective component (P-arm) which was the intervention group, to which MDR TB patients were enrolled onto CB DOT, and the retrospective component (R-arm) as the comparison group consisting of DR TB patients who received HF DOT.

#### Prospective arm

A CB DOT intervention developed in an earlier study was assigned to eligible MDR-TB patients consecutively [14]. Patients were followed up prospectively until treatment outcomes were determined in accordance with the national guidelines for managing drug-resistant TB [15]. These outcomes included either cure, completion of treatment, treatment failure, death, loss to follow up or administrative censoring of patients. The censor date was set at 30^th^ March 2022.

#### Retrospective arm

The comparison group was comprised of MDR-TB patients who were on HF DOT. These patients had begun their treatment within the year before the study's start date and their treatment outcomes had been assigned by September 30, 2020, which was three months prior to the study's initiation. Data for this group was collected retrospectively from the five DR TB treatment health facilities, including patients’ characteristics at MDR-TB treatment initiation and treatment outcomes. The same data collection tool was used for data collection for both the intervention and comparison groups (see Additional file 2). We adapted a tool based on the diffusion of innovation theory (see additional file 3) to collect data on the acceptability of the CB DOT model [16, 17].

### Study endpoints

#### Primary study endpoint

The primary outcome of interest was treatment success, defined as the sum of MDR patients that were cured or completed treatment.

Cured: defined as completion of treatment as recommended by the national policy without evidence of failure AND 3 or more consecutive cultures taken at least thirty [18] days apart that tested negative after the intensive phase [15].

Treatment completed: defined as completion of treatment as recommended by the national policy without evidence of failure BUT no record that 3 or more consecutive cultures taken at least 30 days apart were negative after the intensive phase [15].

#### Secondary study endpoints

These comprised of other outcomes that included treatment failure, death and lost to follow up.

Treatment failure: refered to treatment termination or need for permanent regimen change of at least 2 anti TB drugs because of 1) lack of conversion by end of the intensive phase, OR 2) Bacteriological reversion in the continuation phase after conversion to negative, OR 3) Evidence of additional acquired resistance to fluoroquinolones or second-line injectable drugs, OR 4) Adverse drug reactions (ADRs) [15].

Died: An MDR patient who dies for any reason during the course of treatment [15].

Lost to follow-up: A patient whose treatment was interrupted for 2 consecutive months or more [15].

### Study setting

A multi-site study at five tertiary referral hospitals purposively selected which accounted for at least 80% of MDR cases enrolled in care in the country and were representative of the major regions of Uganda.

### Selection of community health workers to support community-based DOT

The use of trained lay providers in the provision of DOT for DR TB patients is recommended by the programmatic management of drug-resistant tuberculosis (PMDT) guidelines in Uganda [9, 15]. Previously, with regimens that included injectables, DOT was only recommended for provision by lay providers after the completion of the intensive phase that was characterized by injectables (Kanamycin, Amikacin, or Capreomycin) [15]. However, the dawn of all oral medications makes the provision of CB DOT throughout the treatment cycle for MDR TB patients possible [9, 19, 20]. We selected lay providers who were; 1) chosen by or acceptable to the patient 2) committed to supporting the patient throughout their treatment 3) able to read and write 4) not an immediate family member [14, 15] 5) able to accompany the patient to the clinic for monthly appointments 6) had previously supported health initiatives at the community level including TB and MDR TB 7) of good standing in the community and 8) living close to the patient and not take him or her more than an hour to reach the patient’s home. We planned that each community health worker (CHW) was to support daily DOT up to a maximum of three DR TB patients if within the same locality for efficiency and ease of administration. CHWs were trained on CB DOT model implementation, their role in supporting the DR TB patient, and DR TB specific training that focused on basic TB, TB classifications, the definition of DR TB, causes of DR TB, clinical presentation, medicines used, associated side effects, and drug storage. Ongoing support was availed to CHWs during monthly routine visits to the tertiary referral hospital. This ongoing support, facilitated by the study clinician, was instrumental toward enhancing the CHWs' proficiency in effectively carrying out their roles and ensuring the successful implementation of the CB DOT model for DR TB management.

### Roles of CHWs

The roles of the CHWs included; 1) Conduct daily visits to the patient’s home to observe him/her swallowing the drug, 2) Record daily community DOT in the patient follow-up log that was presented at the DR TB treatment initiation facility every month 3) Observe during daily interaction with the patient and record any adverse events reported by the patient, any concerns or actions taken 4) Consult virtually with the site study focal person/ clinician on an ongoing basis on all aspects of care, including liaising with initiating facilities on the management of adverse events, including referrals 5) Remind patients of monthly health facility visits 6) Pick up drugs for MDR-TB patients monthly 7) Ensure safe custody/keeping of drugs in a drug box away from dust at room temprature 8) Provide patient health education and infection prevention and control messages to patients and members of the family about MDR- TB, and 9) Provide **a**dherence counseling.

### Selection of study participants

#### Sample size determination

The total number of DR-TB patients in care in the five health facilities by 2019 was 302 patients. Based on the 2018 /19 Uganda National Tuberculosis and Leprosy Program (NTLP) report, the approximate treatment success rate among MDR-TB patients in Uganda was 75%. Assuming a statistical power of 80%, a two-sided hypothesis test with a 5% significance level (standard normal deviate at 95% confidence, Z=1.96). The desired sample size to detect a 10% increase in treatment success due to CB DOT to achieve the national target of treatment success of 85% was 185 patients in each arm. The estimatedsample size adjusted using finite population correction because of the limited available numbers of DR patients [21]. Sampling proportionate to size was used to determine the number of study participants selected from each of the participating hospitals.

#### Study inclusion criteria

DR TB patients above 18 years of age on a standard short treatment regimen (sSTR) [15] who had completed the injectable phase of DR TB treatment, or those on an all oral modified STR (mSTR) [6] or, on long all oral regimens [6] at the point of enrolment onto the study, after providing signed informed consent, were willing and able to comply with scheduled visits, treatment plan, laboratory tests, and other procedures relevant to assigning treatment outcomes.

#### Study exclusion criteria

DR TB patients who are on other individualized regimens that included injectables.

### Data collection

For the R-arm, retrospective data were extracted from DR-TB registers at the study hospitals using pre-designed data collection tools. For the *P-arm*, prospective data were collected by research assistants (RAs) using the same tool. Patients received daily visits by the CHWs, who observed medicine ingestion, noted any patient concerns and adverse events, and documented data in a patient log.

### Study variables

The main study outcome was the proportion of patients who registered treatment success (those who were cured or those who completed treatment) among those intiated DR TB treatment. The main comparison groups were DOT models of care, i.e. health facility based DOT (HF DOT) versus community-based DOT (CB DOT).

Other participants’ information collected include; Patient demographics (e.g. age, gender), occupation, high-risk populations, duration of MDR TB treatment and presence of co-infections (e.g. HIV).

### Statistical analysis

Quantitative analyses were conducted using STATA software version 16.1 (StataCorp, College Station, Texas, USA). We described participants’ socio-demographic characteristics for both HF DOT and CB DOT groups using descriptive summaries; frequency and percentages for categorical variables, median and inter-quartile range for count data, i.e. age and duration of MDR-TB treatment.

We use Pearson Chi-square test for categorical variables and Wilcoxon rank-sum test for continuous non-normal variables comparing HF DOT versus CB DOT groups. We used prevalence ratios and 95% confidence intervals to compare treatment success across HF DOT versus CB DOT groups using Poisson regression models with cluster-robust standard errors to account for intra-cluster correlation of participants within a hospital. The analysis was adjusted for participants’ characteristics. Modified Poisson regression was used because it estimates adjusted relative risk or prevalence ratios appropriately when the outcome is common (i.e. prevalence>10%) compared to logistic regression, and it does not have model convergence challenges unlike log-binomial models [22]. Only factors with *P*<0.3 at unadjusted models were included in the multivarible model, except CB DOT type, the main comparison variable. Joint P-values using Wald test at unadjusted were used in the selection of variables with more than two categories in the adjusted model. We checked whether the effect of DOT type was modified by other participants’ characteristics, by fitting interactions between DOT type and MDR-TB treatment type, sex, and age groups was carried out.

### Ethics approval and consent to participate

This research was performed in accordance with the Declaration of Helsinki guidelines. It was approved by the Joint Clinical Research Centre (JC1519) Institutional Review Board (IRB), and by the Uganda National Council of Science and Technology (HS2684) prior to study conduct. Administrative permission to collect this data were provided by the Ministry of Health- National TB and Leprosy Program and by respective study sites. Prior to interview commencement, written informed consent was voluntarily obtained from all participants. Confidentiality of patients was ensured by using study identification numbers and data storage protection procedures.

## Results

**Fig. 1:**
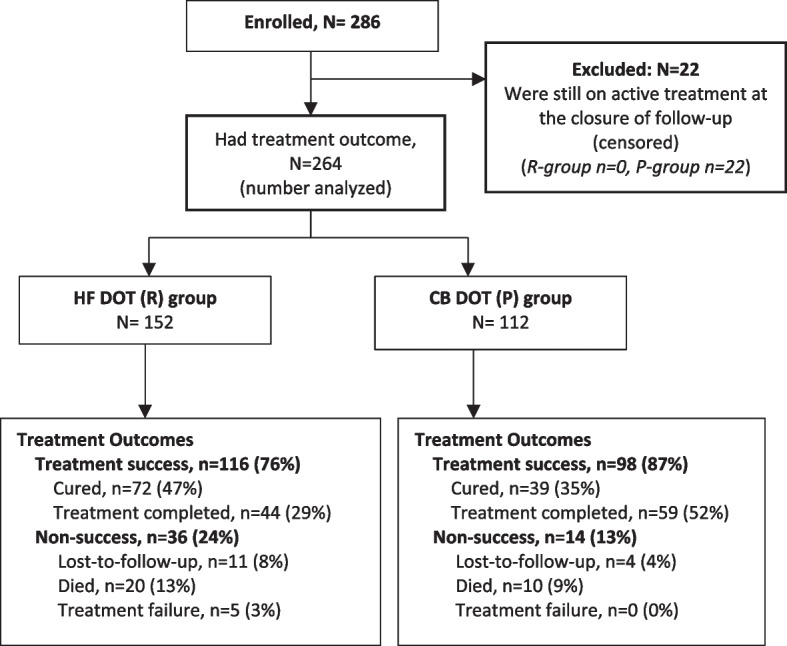
Patient flow diagram

From December 2020 to March 2022, a total of 286 MDR-TB patients were enrolled in the study, 22 were excluded because they were still on active treatment at time of follow-up closure (Table S1) (see Additional file 1). Of the 264 patients that remained, 152 patients were in the HF DOT group (R) and 112 patients in the CB DOT group (P) *(*Fig. 1*).* Majority were males 67.8% with a median age of 36 years (inter-quartile range 29 to 44 years). The distribution of sex, marital status and income were similar across the comparison groups, except for educational level, regimen type and organizational unit. Age was borderline with a *P*-value of 0.05. The CB DOT group had a larger proportion of patients who had attained at least primary education (95% vs 91%). Daily income data was unavailable in the retrospective group (i.e. HF DOT group) (Table 1)*.*

**Table 1:** Participants’ characteristics

	**HF DOT (R-Pre)**	**CB DOT (P-Post)**	**Total**^**a**^	***P*-value**^**b**^
	**(*N* = 152) n (%)**	**(*N* = 112) n (%)**	**(*N* = 264) n (%)**	
**Sex**				0.42
Female	52 (34.2)	33 (29.5)	85 (32.2)	
Male	100 (65.8)	79 (70.5)	179 (67.8)	
**Age (years) at enrollment, median (IQR)**	36.0 (29.0, 42.5)	35.5 (27.0, 45.0)	36.0 (29.0, 44.0)	0.67
**Age (years) at enrollment**^**c**^				0.05
20_24	11 (7.2)	18 (16.1)	29 (11.0)	
25_34	57 (37.5)	32 (28.6)	89 (33.7)	
35_44	52 (34.2)	32 (28.6)	84 (31.8)	
45+	32 (21.1)	30 (26.8)	62 (23.5)	
**Marital status** ^**d**^				0.28
Single	83 (55.3)	54 (48.6)	137 (52.5)	
Married	67 (44.7)	57 (51.4)	124 (47.5)	
**Daily income (USD)**^**d**^				0.26
<$2	1 (50.0)	40 (47.1)	41 (47.1)	
$2 to 5	0 (0.0)	18 (21.2)	18 (20.7)	
$5 to 10	0 (0.0)	19 (22.4)	19 (21.8)	
<$10	1 (50.0)	8 (9.4)	9 (10.3)	
**Educational level**^**d**^				0.04
None	7 (9.1)	5 (4.9)	12 (6.7)	
Primary	36 (46.8)	60 (58.3)	96 (53.3)	
Secondary	16 (20.8)	28 (27.2)	44 (24.4)	
Tertiary	18 (23.4)	10 (9.7)	28 (15.6)	
**BMI<18.5 kg/m**^**2 d**^				0.95
No	84 (60.0)	62 (59.6)	146 (59.8)	
Yes	56 (40.0)	42 (40.4)	98 (40.2)	
**MDR treatment Regimen** ^**d**^** (sSTR/mSTR)?**				< 0.001
No	9 (5.9)	52 (46.4)	61 (23.1)	
Yes	143 (94.1)	60 (53.6)	203 (76.9)	
**Any underlying co-morbidities**^**f**^				0.16
No	75 (49.3)	65 (58.0)	140 (53.0)	
Yes	77 (50.7)	47 (42.0)	124 (47.0)	
**Comorbidity (HIV)**				0.29
No	81 (53.3)	67 (59.8)	148 (56.1)	
Yes	71 (46.7)	45 (40.2)	116 (43.9)	
**Comorbidity (hypertension)**				0.39
No	151 (99.3)	110 (98.2)	261 (98.9)	
Yes	1 (0.7)	2 (1.8)	3 (1.1)	
**Comorbidity (diabetes)**				0.75
No	150 (98.7)	111 (99.1)	261 (98.9)	
Yes	2 (1.3)	1 (0.9)	3 (1.1)	
**Are concomitant medications being taken?**^**g**^				0.42
No	72 (47.7)	57 (52.8)	129 (49.8)	
Yes	79 (52.3)	51 (47.2)	130 (50.2)	
**Concomitant medications (ARVs)**^**h**^				0.51
No	11 (15.5)	5 (11.1)	16 (13.8)	
Yes	60 (84.5)	40 (88.9)	100 (86.2)	
**Concomitant medications (hypertension)**				0.39
No	151 (99.3)	110 (98.2)	261 (98.9)	
Yes	1 (0.7)	2 (1.8)	3 (1.1)	
**Organization unit**				0.03
Hoima RR Hospital	22 (14.5)	6 (5.4)	28 (10.6)	
Lira RR Hospital	25 (16.4)	33 (29.5)	58 (22.0)	
Mbale RR Hospital	17 (11.2)	14 (12.5)	31 (11.7)	
Mubende RR Hospital	11 (7.2)	6 (5.4)	17 (6.4)	
Mulago national referral Hospital	77 (50.7)	53 (47.3)	130 (49.2)	

*HF DOT* denotes Health Facility Directly Observed Treatment, *CB DOT* denotes Community Based (community health workers or family member) Directly Observed Treatment, *sSTR* /mSTR denotes standardized short-term regimen (i.e. in R group) or modified short-term regimen (i.e. in R group). RR denotes Regional Referral Hospital

Antiretroviral therapy (ARV) is indicated only to the HIV positives, so denominator reduces, *N* = 116 (HF DOT group *n* = 71, CB DOT group *n* = 45)

^a^Analysis restricted to only those who had treatment outcomes at the time of analysis

^b^*P*-values from the Chi-square test for categorical variables and Wilcoxon rank-sum test for continuous age comparing HF DOT versus CB DOT groups

^c^Age categorized as per the 2016 Uganda Demographic and Health Survey

^d^Missing values: Daily income (*n* = 177, 67%), educational level (*n* = 84, 32%), body mass index (*n* = 20, 8%), marital status (*n* = 3, 1%), MDR treatment regimen (*n* = 4, 1.5%)

^e^Elementary occupations consist of simple and routine tasks which mainly require the use of hand-held tools and often some physical effort (require skill at the first skill level)

^f^Any comorbidity included: HIV, hypertension, and diabetes

^g^Any concomitant medication included: antiretroviral drugs, anti-hypertensive drugs, and anti-diabetic drugs

Treatment success was significantly higher in the CB DOT group compared to the HF DOT group (88% vs 76%, Chi-square test *P* value=0.01). Those who were loss to follow up were twice as high in the HF DOT group (7.2%) compared to the CB DOT group (3.6%). Additionally, more death (13.2% Vs 8.9%) and treatment failures (3.3% Vs 0.0%) were recorded among those on the HF DOT compared to CB DOT group (Table 2)*.*

**Table 2:** Treatment outcome by MDR-TB DOT type (unadjusted)

	**HF DOT (R-Arm)** *N* = 152 n (%)	**CB DOT (P-Arm)** *N* = 112 n (%)	**Total** *N* = 264 n (%)	***P*-value†**
**Treatment success**	**116 (76.3)**	**98 (87.5)**	**31 (73.8)**	**0.01**
^**a**^**Treatment non-success**	**36 (23.7)**	**14 (12.5)**	**11 (26.2)**	
*Loss to Follow up*	*11 (7.2)*	*4 (3.6)*	*15 (5.7)*	
*Died*	*20 (13.2)*	*10 (8.9)*	*30 (11.4)*	
*Treatment Failure*	*5 (3.3)*	*0 (0.0)*	*5 (1.9)*	

*DOT* denotes Directly Observed Therapy, *HF DOT* denotes Health facility DOT, *CB DOT* community-based DOT

^†^ *P*-value comparing treatment success across HF DOT versus CBDOT groups adjusted for intra-cluster correlation of patients’ outcome within a hospital using unadjusted Poisson regression with cluster-robust standard errors

^a^Treatment non-success 36 (as a sum of those who were loss to follow up, died or had treatment failure). The percentage of those who died, loss to follow-up or treatment failure is out of the total number in each arm

Treatment success in the CB DOT group was 12%-points higher compared to that in the HF DOT group, after adjusting for the patient’s sex, age group, marital status, employment status, DR TB regimen duration type, and whether a patient had one or more comorbidities (adjusted prevalence ratio 1.12, 95%CI 1.01, 1.24, *P* value=0.03). Males were less likely to achieve treatment success compared to their female counterparts (aPR=0.87, 95%CI 0.78, 0.98, *P* value=0.02). Whereas age groups, marital status, employment status, DR-TB regimen duration type, and having a comorbidity were not significantly associated with DR-TB treatment success. There was no significant effect modification of the effect of DR-TB DOT type due to other participants’ characterisitics (Table 3)*.*

**Table 3:** Association between DOT type and MDR-TB treatment success, adjusting for other patients’ characteristics

Factors	No.of patients in a group, *N* = 264 N	Treatment success n (%)^a^	Un adjusted Prevalence Ratio (95%CI)	*P* value	Adjusted Prevalence Ratio (95%CI)^b^	*P* value
**MDR-TB DOT type**						
HF-DOT group	152	116 (76.3)	1		1	
CB-DOT group	112	98 (87.5)	1.15 (1.03, 1.27)	0.01	1.12 (1.01, 1.24)	0.03
**Sex**						
Female	85	75 (88.2)	1		1	
Male	179	139 (77.6)	0.88 (0.77, 1.00)	0.05	0.87 (0.78, 0.98)	0.02
**Age group (years) at enrollment**^**c**^						
20 – 24	29	25 (86.2)	1		1	
25 – 34	89	75 (84.3)	0.98 (0.84, 1.44)	0.77	1.04 (0.97, 1.12)	0.25
35 – 44	84	65 (77.4)	0.90 (0.81, 1.00)	0.05	0.96 (0.88, 1.05)	0.41
≥ 45	62	49 (79.0)	0.92 (0.83, 1.02)	0.10	0.97 (0.91, 1.03)	0.34
**Marital status**						
Single	137	112 (81.7)	1		1	
Married	124	99 (79.8)	0.98 (0.87, 1.10)	0.68	0.89 (0.70, 1.13)	0.33
**Body mass index<18.5 kg/m**^**2**^						
No	146	122 (83.6)	1			
Yes	98	74 (75.5)	0.90 (0.64, 1.27)	0.56	**-**	
**Patient was on sSTR/mSTR MDR-TB regimen type**						
No	61	53 (86.9)	1		1	
Yes	203	161 (79.3)	0.91 (0.84, 0.99)	0.04	0.95 (0.85, 1.06)	0.36
**Any comorbidity**^**f**^						
No	140	120 (85.7)	1		1	
Yes	124	94 (75.8)	0.88 (0.75, 1.05)	0.15	0.88 (0.76, 1.02)	0.10
**HIV co-infection**						
No	148	125 (84.5)	1			
Yes	116	89 (76.7)	0.91 (0.78, 1.06)	0.23	**-**	
**Hypertension comorbidity**						
No	261	212 (81.2)	**-**		**-**	
Yes	3	2 (66.7)	**-**		**-**	
**Hypertension diabetes**						
No	261	213 (81.6)	**-**		**-**	
Yes	3	1 (33.3)	**-**		**-**	
**Any concomitant medication**^**g**^						
No	129	111 (86.1)	1			
Yes	130	98 (75.4)	0.88 (0.75, 1.05)	0.15		
**ARVs co-medication**						
No	16	13 (81.3)	**-**		**-**	
Yes	100	76 (76.0)	**-**		**-**	

N denotes the total number of patients in a category of a specific patient’s characteristic, and **n** denotes the number of patients with treatment success

Only factors with *P* < 0.3 were included in the adjusted model except CB DOT type the main comparison variable. Joint Wald P-values at unadjusted were used in the selection of variables with more than 2 categories into the adjusted model (age groups, joint *P* = 0.01). Checking for effect modification on the effect of MDR-TB DOT type, interactions between DOT type and MDR-TB treatment type, sex, and age groups were not significant (*P*-values: 0.11, 0.38, 0.39, respectively)

^a^Percent (%) treatment success was computed as the number of patients with treatment success (n) out of the total number of patients (N) in each characteristic category

^b^Prevalence ratios were estimated from the modified Poisson model with robust cluster variances to account for the intra-cluster correlation of participants within a hospital. No covariate in the adjusted model had missing values (*N* = 264)

To further understand participant characteristics by sex, it was found that age (*P*-value=0.036), median age of 36 (IQR 29–44; P-value 0.004) and occupation (*P*-value =0.003) were statistically significant (Table S3) (see Additional file 1).

A total of 126 (47.7%) out of 264 patients reported at least one adverse event. The HF DOT group had a higher proportion of patients with at least one adverse event compared to the CB DOT group (90/152 [59.2%] versus 36/112 [32.1], *P*-value<0.01) (Table 4)*.*

**Table 4:** Adverse events profile by study groups

Adverse drug reaction	Overall n/N (%)	HF DOT (R group) n/N (%)	CB DOT (P group) n/N (%)	*P*-value
Patients with any adverse event	126/264 (47.7)	90/152 (59.2)	36/112 (32.1)	< 0.01
Total adverse event	687	481	206	

The most common adverse events were gastrointestinal disorders (18.9%), flu-like symptoms (14.6%), musculoskeletal and connective tissue disorders (13.0%), general disorders and administration site conditions (12.8%), and nervous system disorders (12.8%) (Table S2) (see Additional file 1).

The model demonstrated widespread acceptability, with over 90% acceptance among both patients (Table S4) and health workers (Table S5) (see Additional file 1)*.*

## Discussion

We carried out a quasi-experimental pre and post-study to assess the effectiveness of a community-based model for the management of MDR-TB. Retrospective data were collected for patients who had received care under the HF DOT model a year before the study commenced. We found that CB DOT increased treatment success by 12-percentage points compared to the HF DOT group. Males were less likely to complete treatment compared to their female counterparts while other factors like age, marital status, employment status, and having any comorbidity were not significantly associated with DR TB treatment success. However, higher proportions of at least one adverse event was reported in HF DOT group compared to the CB DOT group. The model was found to be highly acceptable among DR-TB patients and health workers. Further, as regards to secondary outcomes (loss to follow up, death and treatment failure) findings were more favourable among those on CB DOT compared to the HF DOT.

There is evidence from earlier studies for better treatment outcomes for MDR-TB patients managed using community-based models in Sub-Saharan Africa and Asia [12, 20, 23, 24]. The increase in treatment success found in our study (12%) was comparable to that from a cohort study that engaged CHWs to provide home-based care for DR-TB patients in rural Eswatini (11%) [25]. Improvements in treatment success documented by community-based models are probably due to the convenience, decrease in care costs associated with home-based care [26] and lower rates of side effects associated with regimen type.

Globally, the male sex is disproportionately affected by TB [27–29]. Men are also more likely to have suboptimal treatment outcomes [12, 18, 29–31]. Further, similar results were found among men in a community-based DOT model for the management of MDR-TB carried out in South Africa [32]. Men were more likely to suffer from TB than women due to behavioral factors like smoking [33], alcohol use disorder and drug abuse as well as occupational harzards (mining, construction and transportation). In our study, we observed that men had an average age that was five years older than women. Our analysis of patient characteristics based on sex revealed a statistically significant association with occupation. Notably, the transport sector was predominantly composed of men.

In our study, age was not significantly associated with treatment outcomes. This is similar to findings from a systematic review and meta- analysis on treatment outcomes for DR-TB patients on a community-based model of care of studies on found that age was not a significant contributor to treatment outcomes irrespective of the model of care [23]. However, in some instances, advanced age has been found to be significantly associated with unfavorable outcomes among MDR-TB [34–36]. The findings in our study could be explained by the fact that the majority (78.9%) of the study participants were less than 45 years of age.

Social factors such as being married, being employed, and having social support have been documented to positively affect treatment outcomes among MDR-TB patients [37]. These social factors were not significantly associated with successful treatment in this study and could be explained by the fact that Uganda NTLP implements an enabler program that provides all DR TB patients with dry rations and transport vouchers to facilitate monthly visits to tertiary hospitals for treatment monitoring, preventing their households from experiencing catastrophic costs [38]. Transportation is a well documented barrier during chronic care among vulnerable populations [39]. While interventions that overcome transportation barriers can result into better outcomes as demonstrated in our study, ensuring sustainability of these approaches is challenging in low resource settings, and yet are essential for continued positive outcomes.

Comorbidities, including HIV, diabetes, and hypertension have been documented to contribute to poor treatment outcomes among DR-TB patients [40–42]. Despite the fact that those infected with HIV showed a tendency towards poorer outcomes, our study did not find underlying comorbidities to be statistically significantly associated with treatment success. This could be have been due to small numbers in this category.

Similar to our study, lower rates of adverse events have been reported in other studies using community-based models of care for DR TB, it is possible that adverse events could have been underreported by CHWs [43]. A systematic review carried out in 2016 showed that the most common adverse events were GIT [44], similar to our study. Suspected agents that cause GIT adverse reactions are majorly caused by ethionamide (Eto), bedaquiline (BDQ), and clofazimine (Cfz) [11] which are part of the available DR TB treatment regimens. Our study reported lower rates of ototoxicity due to a 2019 policy change in Uganda that called for a shift to all oral regimens with avoidance of injectables such as kanamycin likely to cause ototoxicity [6]. Majority of patients on the R-arm were on injectable based regimens that were associated with more side effects compared to all oral regimens. It is possible that the lower rates of side effects in the P-arm compared to the R-arm was due to predominant regimen type other than the mode of delivery.

Further, community-based models for the management of MDR-TB have been documented to be acceptable among patients, CHWs, and health care workers elsewhere including in Uganda [14].

This study had many strengths. We included regional representation and hence findings can be generalized to all MDR-TB patients across the country. Further studies using rigorous study designs like randomized controlled trials are recommended. Study limitations included the fact that COVID-19 related travel restrictions could have led to lower treatment completion or loss to follow up in the CB DOT group than would have been observed in the absence of travel restrictions. The pre-post-study design has some limitations associated with temporal changes. During the course of study implementation, Uganda updated its guidelines for management of DR TB and phased out the use of injectables (sSTR), during the analysis this was adjusted for, and we found no difference in outcomes between those who were on sSTR compared to those on mSTR.

The R arm had some missing variables, for example income, making comparisons between the two groups difficult. Lastly, we extracted data on the type of regimen a patient was on that was either sSTR or mSTR or other individualized regimens. We did not extract data on the individual DR-TB drug molecules (notably among those with individualized regimens), making it difficult to assess whether any differences in specific drug molecules used among the two groups could affect the outcome. Forexample individual drug molecules like linezolid have been associated with poor treatment outcomes among patients with DR TB [45].

## Conclusions

CB DOT for MDR-TB care is effective and results in better treatment outcomes than HF DOT. Community-based services for MDR-TB care should be adopted by the NTLP on a wider scale as it brings the services of care closer to the people. The use of CHWs to complement service delivery for MDR-TB is possible and can lead to improved patient outcomes. However, there is a need for more research to better understand the role of CHWs in the care of patients with MDR-TB and to develop effective models for their integration into the healthcare system. Further, the cost-effectiveness of this model of care should be further evaluated.

### Recommendations for practice and policy

- Develop national guidelines for the recruitment, training, and deployment of CHWs in the management of MDR-TB. It is critical to ensure that CHWs receive adequate training and supervision to provide effective and safe care to DR TB patients.
- Integrate CHWs into the existing healthcare system and ensure that they work in collaboration with healthcare providers. This can help to improve communication and coordination of care, which is essential for successful treatment outcomes.
- Monitor and evaluate the effectiveness of CHW programs and foster partnerships between government, implementing partners and other stakeholders to support the implementation of CHW programs for sustainability.

### Supplementary Information

**Additional file 1:** **Table S1.** Participants’ characteristics by censored (excluded from analysis) vs not censored. **Table S2.** Adverse events profile (detailed). **Table S3.** Participants characteristics by sex. **Table S4.** Acceptability and adaptation of community-based MDR-TB DOT program – patients’ perception. Table S5: Acceptability and adaptation of community-based MDR-TB DOT program – providers’ perception.

**Additional file 2.**

**Additional file 3.**

## Data Availability

The datasets used and/or analyzed during the current study are available from the corresponding author on request.

## Abbreviations

AE: Adverse event
BDQ: Bedaquiline
CB-DOT: Community-Based Directly Observed Therapy
CEA: Cost Effectiveness Analysis
CHW: Community health worker
Cfz: Clofazimine
Cs: Cycloserine
DLM: Delaminid
DOTS: Directly Observed Therapy Short Course
DOT: Directly Observed Therapy
DST: Drug Susceptibility Testing
GCP: Good Clinical Practice
FPC: Finite Population Correction
HCW: Healthcare Worker
HF-DOT: Health Facility Directly Observed Therapy
HIV: Human Immunodeficiency Virus
IDI: Infectious Diseases Institute
INH: Isoniazid
IRB: Institutional Review Board
Lfx: Levofloxacin
LZD: Linezolid
MDR-TB: Multidrug Resistant TB
mSTR: Modified short treatment regimen
MoH: Ministry of Health
MTB: Mycobacterium Tuberculosis
NRH: National Referral Hospital
NTLP: National TB and Leprosy Program
P-arm: Prospective arm
R-arm: Retrospective arm
RRH: Regional Referral Hospital
SAE: Serious Adverse Event
SMS: Short Message Service
sSTR: Standard Short Term Regimen
SRC: Scientific Review Committee
TB: Tuberculosis
URC: University Research Co., LLC
USAID: United States Agency for International Development
WHO: World Health Organization
