# Supplementary material for: Community-based directly observed therapy is effective and results in better treatment outcomes for patients with multi-drug resistant tuberculosis in Uganda
Source: BMC Health Serv Res. 2023 Nov 13;23:1248. doi: 10.1186/s12913-023-10120-7 (PMC10644403; doi:10.1186/s12913-023-10120-7)
Supplement: Supplementary file 1 — Additional file 1: Table S1. Participants’ characteristics by censored (excluded from analysis) vs not censored. Table S2. Adverse events profile (detailed). Table S3. Participants characteristics by sex. Table S4. Acceptability and adaptation of community-based MDR-TB DOT program – patients’ perception. Table S5: Acceptability and adaptation of community-based MDR-TB DOT program – providers’ perception. [file 12913_2023_10120_MOESM1_ESM.docx]

**Table S1: Participants’ characteristics by censored (excluded from analysis) vs not censored**

| \|  \| **Not censored** \| **Censored** \|  \| \| --- \| --- \| --- \| --- \| \|  \| **(N = 264)**  **n (%)** \| **(N = 23)**  **n (%)** \| **p-value** \| |
| --- | --- | --- | --- | --- | --- | --- | --- | --- |
| \| **Study group** \|  \|  \| <0.001 \| \| --- \| --- \| --- \| --- \| \| HFDOT (R-Pre) \| 152 (57.6) \| 0 (0.0) \|  \| \| CBDOTS (P-Post) \| 112 (42.6) \| 22 (95.7) \|  \| \| **Sex** \|  \|  \| 0.882 \| \| Female \| 85 (32.2) \| 7 (30.4) \|  \| \| Male \| 179 (67.8) \| 16 (69.6) \|  \| \| **Age (years) at enrollment** \|  \|  \| 0.663 \| \| 20_24 \| 29 (11.0) \| 2 (8.7) \|  \| \| 25_34 \| 89 (33.8) \| 6 (26.1) \|  \| \| 35_44 \| 83 (31.6) \| 7 (30.4) \|  \| \| 45+ \| 62 (23.6) \| 8 (34.8) \|  \| \| **Marital status** \|  \|  \| 0.212 \| \| Single \| 137 (51.9) \| 9 (39.1) \|  \| \| Married \| 124 (47.0) \| 14 (60.9) \|  \| \| Missing \| 3 (1.1) \| 0 (0.0) \|  \| \| **Daily income (USD)** \|  \|  \| 0.333 \| \| <$2 \| 41 (15.5) \| 11 (47.8) \|  \| \| $2 to 5 \| 18 (6.8) \| 4 (17.4) \|  \| \| $5 to 10 \| 19 (7.2) \| 2 (8.7) \|  \| \| >$10 \| 9 (3.5) \| 0 (0.0) \|  \| \| Not stated \| 177 (67.0) \| 6 (26.1) \|  \| \| **Education level** \|  \|  \| 0.308 \| \| None \| 12 (4.5) \| 1 (4.3) \|  \| \| Primary \| 96 (36.4) \| 17 (73.9) \|  \| \| Secondary \| 44 (16.7) \| 3 (13.0) \|  \| \| Tertiary \| 28 (10.6) \| 2 (8.7) \|  \| \| Missing \| 84 (31.8) \| 0 (0.0) \|  \| \| **BMI<18.5 kg/m2** \|  \|  \| 0.017 \| \| No \| 146 (55.3) \| 7 (30.4) \|  \| \| Yes \| 98 (37.1) \| 14 (60.9) \|  \| \| Missing \| 20 (7.6) \| 2 (8.7) \|  \| \| **MDR treatment Regimen (STR/mSTR)?** \|  \|  \| <0.001 \| \| No \| 60 (22.7) \| 16 (69.6) \|  \| \| Yes \| 200 (75.8) \| 7 (30.4) \|  \| \| Missing \| 4 (1.5) \| 0 (0.0) \|  \| \| **Any underlying co-morbidities** \|  \|  \| 0.254 \| \| No \| 140 (53.0) \| 15 (65.2) \|  \| \| Yes \| 124 (47.0) \| 8 (34.8) \|  \| \| **Comorbidity (HIV)** \|  \|  \| 0.204 \| \| No \| 148 (56.1) \| 16 (69.6) \|  \| \| Yes \| 116 (43.9) \| 7 (30.4) \|  \| \| Concomitant Medications being taken? \|  \|  \| 0.213 \| \| No \| 129 (48.9) \| 14 (63.6) \|  \| \| Yes \| 130 (49.2) \| 8 (36.4) \|  \| \| Missing \| 5 (1.9) \| 0 (0.0) \|  \| \|  \|  \|  \|  \| \| Organization unit name \|  \|  \| 0.032 \| \| Hoima RR Hospital \| 28 (10.6) \| 0 (0.0) \|  \| \| Lira RR Hospital \| 58 (22.0) \| 11 (50.0) \|  \| \| Mbale RR Hospital \| 31 (11.7) \| 2 (9.1) \|  \| \| Mubende RR Hospital \| 17 (6.4) \| 2 (9.1) \|  \| \| Mulago National Referral Hospital - Ward 5&6(TB Ward) \| 130 (49.2) \| 7 (31.8) \|  \| |

*^a^22 patients were censored because they were still on treatment*

**Table S2: Adverse events profile (detailed)**

| **AE (preferred term)** | **HFDOT (R-group)**  **number** | **CBDOTS (P-group)**  **number** | **Total**  **number** |
| --- | --- | --- | --- |
| Joint pain | 43 | 24 | 67 |
| Neuropathy | 27 | 25 | 52 |
| Vomiting | 37 | 3 | 40 |
| Chest pain | 35 | 4 | 39 |
| Epigastric pain | 22 | 9 | 31 |
| Cough | 19 | 8 | 27 |
| Weakness generalized | 17 | 7 | 24 |
| Appetite lost | 15 | 8 | 23 |
| Visual disturbance | 13 | 7 | 20 |
| Difficulty breathing | 8 | 11 | 19 |
| Hearing losses | 17 | 0 | 17 |
| Nausea | 16 | 1 | 17 |
| Palpitations | 10 | 6 | 16 |
| Headache | 9 | 6 | 15 |
| Oedema | 6 | 9 | 15 |
| Pruritus | 12 | 1 | 13 |
| Dizziness | 4 | 7 | 11 |
| Fever | 9 | 2 | 11 |
| Back ache | 9 | 1 | 10 |
| Rash | 4 | 6 | 10 |
| Conjunctivitis | 4 | 4 | 8 |
| Weight loss | 5 | 3 | 8 |
| Fatiguability | 5 | 2 | 7 |
| Haemoptysis | 7 | 0 | 7 |
| Tinnitus | 7 | 0 | 7 |
| Abdominal pain | 4 | 2 | 6 |
| Abdominal pain lower | 5 | 1 | 6 |
| Hyperglycaemia | 6 | 0 | 6 |
| Myalgia | 0 | 6 | 6 |
| Unspecified psychosis | 3 | 3 | 6 |
| Anaemia | 2 | 3 | 5 |
| Dysuria | 4 | 1 | 5 |
| Electrocardiogram Q.. | 3 | 2 | 5 |
| Constipation | 3 | 1 | 4 |
| Coryza | 3 | 1 | 4 |
| Diarrhoea | 4 | 0 | 4 |
| Dysphagia | 2 | 2 | 4 |

**Table S2 (continued)**

| **AE (preferred term)** | **HFDOT (R-group)**  **number** | **CBDOTS (P-group)**  **number** | **Total**  **number** |
| --- | --- | --- | --- |
| Frequency of mictur.. | 3 | 1 | 4 |
| Skin discolouration | 2 | 2 | 4 |
| Dehydration | 1 | 2 | 3 |
| Insomnia | 3 | 0 | 3 |
| Pleurisy | 2 | 1 | 3 |
| Rash pruritic | 3 | 0 | 3 |
| Sore mouth | 2 | 1 | 3 |
| Abdominal distension | 1 | 1 | 2 |
| Ear infection | 2 | 0 | 2 |
| Gastritis | 2 | 0 | 2 |
| Generalised aching | 1 | 1 | 2 |
| Genital ulcer syndr.. | 2 | 0 | 2 |
| Gynaecomastia | 2 | 0 | 2 |
| Hearing disorders | 0 | 2 | 2 |
| Hemiplegia | 2 | 0 | 2 |
| Loose stools | 2 | 0 | 2 |
| Night sweats | 1 | 1 | 2 |
| Odynophagia | 2 | 0 | 2 |
| Palatal pigmentation | 1 | 1 | 2 |
| Pelvic inflammatory.. | 2 | 0 | 2 |
| Soft tissue injury | 0 | 2 | 2 |
| Sore throat | 1 | 1 | 2 |
| Tachycardia | 2 | 0 | 2 |
| Tachypnoea | 0 | 2 | 2 |
| Toothache | 2 | 0 | 2 |
| Urinary tract infec.. | 1 | 1 | 2 |
| Vaginal discharge | 1 | 1 | 2 |
| Anal ulcer | 1 | 0 | 1 |
| Anxiety | 0 | 1 | 1 |
| Ascites | 1 | 0 | 1 |
| Bed sore | 1 | 0 | 1 |
| Body temperature in.. | 1 | 0 | 1 |
| Bowel incontinence | 1 | 0 | 1 |
| Crust | 1 | 0 | 1 |
| Death | 1 | 0 | 1 |
| Decreased appetite | 1 | 0 | 1 |

**Table S2 (continued)**

| **AE (preferred term)** | **HFDOT (R-group) number** | **CBDOTS (P-group) number** | **Total number** |
| --- | --- | --- | --- |
| Depression | 0 | 1 | 1 |
| Drowsiness | 1 | 0 | 1 |
| Dry skin | 0 | 1 | 1 |
| Dysarthria | 1 | 0 | 1 |
| Eruption | 1 | 0 | 1 |
| Excess sweating | 1 | 0 | 1 |
| General body pain | 1 | 0 | 1 |
| Gluteal abscess | 1 | 0 | 1 |
| Haematemesis | 1 | 0 | 1 |
| Haemorrhoids | 1 | 0 | 1 |
| Headaches | 1 | 0 | 1 |
| Hypoxia | 1 | 0 | 1 |
| Intracranial space .. | 1 | 0 | 1 |
| Jaundice | 1 | 0 | 1 |
| Lung fibrosis | 0 | 1 | 1 |
| Lymphopenia | 0 | 1 | 1 |
| Malaise | 1 | 0 | 1 |
| Malnutrition | 0 | 1 | 1 |
| Neck stiff | 0 | 1 | 1 |
| Neutropenia | 0 | 1 | 1 |
| Numbness | 1 | 0 | 1 |
| Oesophageal candidi.. | 1 | 0 | 1 |
| Oral candidiasis | 1 | 0 | 1 |
| Orchitis | 1 | 0 | 1 |
| Orthopnoea | 1 | 0 | 1 |
| Pain in jaw | 1 | 0 | 1 |
| Paraplegia | 0 | 1 | 1 |
| Polyuria | 1 | 0 | 1 |
| Pulmonary congestion | 1 | 0 | 1 |
| Scar | 1 | 0 | 1 |
| Skin scaly | 1 | 0 | 1 |
| Sneezing | 1 | 0 | 1 |
| Stroke | 1 | 0 | 1 |
| Tearing eyes | 1 | 0 | 1 |
| Vaginal itching | 1 | 0 | 1 |
| Vulval wart | 0 | 1 | 1 |
| Warts | 1 | 0 | 1 |
| Weakness of limbs | 1 | 0 | 1 |
| Weight gain | 1 | 0 | 1 |
| Wheezing | 1 | 0 | 1 |
| Total AEs | 481 | 206 | 687 |

**Table S3: Participants characteristics by sex**

| \|  \| Female \| Male \| Total \|  \| \| --- \| --- \| --- \| --- \| --- \| \|  \| (N = 85) \| (N = 179) \| (N = 264) \| p-value \| |
| --- | --- | --- | --- | --- | --- | --- | --- | --- | --- | --- |
| \| **Age (years) at enrollment** \|  \|  \|  \| 0.004 \| \| --- \| --- \| --- \| --- \| --- \| \| Median (Q1, Q3) \| 32.0 (27.0, 41.0) \| 37.0 (31.0, 45.0) \| 36.0 (29.0, 44.0) \|  \| \| **Study goup** \|  \|  \|  \| 0.415 \| \| HFDOT (R-Arm) \| 52 (61.2%) \| 100 (55.9%) \| 152 (57.6%) \|  \| \| CBDOTS (P-Arm) \| 33 (38.8%) \| 79 (44.1%) \| 112 (42.4%) \|  \| \| **Age (years) at enrollment)** \|  \|  \|  \| 0.036 \| \| 20_24 \| 12 (14.1%) \| 17 (9.5%) \| 29 (11.0%) \|  \| \| 25_34 \| 37 (43.5%) \| 52 (29.1%) \| 89 (33.7%) \|  \| \| 35_44 \| 21 (24.7%) \| 63 (35.2%) \| 84 (31.8%) \|  \| \| 45+ \| 15 (17.6%) \| 47 (26.3%) \| 62 (23.5%) \|  \| \| **Marital status** \|  \|  \|  \| 0.300 \| \| Single \| 48 (57.1%) \| 89 (50.3%) \| 137 (52.5%) \|  \| \| Married \| 36 (42.9%) \| 88 (49.7%) \| 124 (47.5%) \|  \| \| **Daily income (USD)** \|  \|  \|  \| 0.280 \| \| <$2 \| 13 (65.0%) \| 28 (41.8%) \| 41 (47.1%) \|  \| \| $2 to 5 \| 3 (15.0%) \| 15 (22.4%) \| 18 (20.7%) \|  \| \| $5 to 10 \| 2 (10.0%) \| 17 (25.4%) \| 19 (21.8%) \|  \| \| >$10 \| 2 (10.0%) \| 7 (10.4%) \| 9 (10.3%) \|  \| \| **Occupation** \|  \|  \|  \| 0.003 \| \| Peasant farmer \| 31 (36.5%) \| 62 (34.6%) \| 93 (35.2%) \|  \| \| Elementary occupations \| 23 (27.1%) \| 31 (17.3%) \| 54 (20.5%) \|  \| \| Service and sales \| 11 (12.9%) \| 20 (11.2%) \| 31 (11.7%) \|  \| \| Professional job \| 4 (4.7%) \| 11 (6.1%) \| 15 (5.7%) \|  \| \| Casual labor \| 2 (2.4%) \| 7 (3.9%) \| 9 (3.4%) \|  \| \| Transport sector \| 0 (0.0%) \| 29 (16.2%) \| 29 (11.0%) \|  \| \| Unemployed \| 10 (11.8%) \| 8 (4.5%) \| 18 (6.8%) \|  \| \| Unknown \| 4 (4.7%) \| 11 (6.1%) \| 15 (5.7%) \|  \| \| **Education** \|  \|  \|  \| 0.573 \| \| None \| 5 (9.3%) \| 7 (5.6%) \| 12 (6.7%) \|  \| \| Primary \| 25 (46.3%) \| 71 (56.3%) \| 96 (53.3%) \|  \| \| Secondary \| 14 (25.9%) \| 30 (23.8%) \| 44 (24.4%) \|  \| \| Tertiary \| 10 (18.5%) \| 18 (14.3%) \| 28 (15.6%) \|  \| \| **BMI<18.5 kg/m2** \|  \|  \|  \| 0.629 \| \| No \| 49 (62.0%) \| 97 (58.8%) \| 146 (59.8%) \|  \| \| Yes \| 30 (38.0%) \| 68 (41.2%) \| 98 (40.2%) \|  \| \| **Regimen type (STR/mSTR)?** \|  \|  \|  \| 0.147 \| \| No \| 15 (17.6%) \| 46 (25.7%) \| 61 (23.1%) \|  \| \| Yes \| 70 (82.4%) \| 133 (74.3%) \| 203 (76.9%) \|  \| \| **Any underlying co-morbidities** \|  \|  \|  \| 0.180 \| \| No \| 40 (47.1%) \| 100 (55.9%) \| 140 (53.0%) \|  \| \| Yes \| 45 (52.9%) \| 79 (44.1%) \| 124 (47.0%) \|  \| \| **HIV** \|  \|  \|  \| 0.217 \| \| No \| 43 (50.6%) \| 105 (58.7%) \| 148 (56.1%) \|  \| \| Yes \| 42 (49.4%) \| 74 (41.3%) \| 116 (43.9%) \|  \| \| **Hypertension** \|  \|  \|  \| 0.199 \| \| No \| 83 (97.6%) \| 178 (99.4%) \| 261 (98.9%) \|  \| \| Yes \| 2 (2.4%) \| 1 (0.6%) \| 3 (1.1%) \|  \| \| **Diabetes** \|  \|  \|  \| 0.230 \| \| No \| 85 (100.0%) \| 176 (98.3%) \| 261 (98.9%) \|  \| \| Yes \| 0 (0.0%) \| 3 (1.7%) \| 3 (1.1%) \|  \| \| **Concomitant Medications being taken?** \|  \|  \|  \| 0.155 \| \| No \| 36 (43.4%) \| 93 (52.8%) \| 129 (49.8%) \|  \| \| Yes \| 47 (56.6%) \| 83 (47.2%) \| 130 (50.2%) \|  \| \| **Concomitant medications (ARVs)** \|  \|  \|  \| 0.115 \| \| No \| 47 (55.3%) \| 117 (65.4%) \| 164 (62.1%) \|  \| \| Yes \| 38 (44.7%) \| 62 (34.6%) \| 100 (37.9%) \|  \| \| **Concomitant medications (hypertension)** \|  \|  \|  \| 0.199 \| \| No \| 83 (97.6%) \| 178 (99.4%) \| 261 (98.9%) \|  \| \| Yes \| 2 (2.4%) \| 1 (0.6%) \| 3 (1.1%) \|  \| \| **Organization unit** \|  \|  \|  \| 0.073 \| \| Hoima RR Hospital \| 9 (10.6%) \| 19 (10.6%) \| 28 (10.6%) \|  \| \| Lira RR Hospital \| 11 (12.9%) \| 47 (26.3%) \| 58 (22.0%) \|  \| \| Mbale RR Hospital \| 14 (16.5%) \| 17 (9.5%) \| 31 (11.7%) \|  \| \| Mubende RR Hospital \| 4 (4.7%) \| 13 (7.3%) \| 17 (6.4%) \|  \| \| Mulago National Referral Hospital \| 47 (55.3%) \| 83 (46.4%) \| 130 (49.2%) \|  \| |

**Table S4: Acceptability and adaptation of community-based MDR-TB DOT program – patients’ perception**

| Items | Strongly Disagree | Somewhat disagree | Disagree | Somewhat agree | Agree | Strongly agree | Total  respondents |
| --- | --- | --- | --- | --- | --- | --- | --- |
| I'm supportive of the community-based MDR-TB DOT program | 1 (2.1) | 0 | 0 | 2 (4.3) | 0 | 44 (93.6) | 47 |
| I don't think the community-based MDR-TB DOT program is good for MDR TB patients | 45 (95.7) | 1 (2.1) | 0 | 0 | 1 (2.1) | 0 | 47 |
| I'm enthusiastic about the community-based MDR-TB DOT program | 0 | 0 | 0 | 4 (8.5) | 0 | 43 (91.5) | 47 |
| I don't expect the community-based MDR-TB DOT program will result in any improvement for MDR TB patients like me | 40 (87.0) | 2 (4.3) | 0 | 1 (2.2) | 0 | 3 (6.5) | 46 |
| The community-based MDR-TB DOT program is very much needed by MDR-TB patients like me | 0 | 0 | 0 | 2 (4.4) | 0 | 44 (95.6) | 46 |
| I don't know what the community-based MDR-TB DOT program is about | 39 (84.8) | 3 (6.5) | 0 | 0 | 0 | 4 (8.7) | 46 |
| The community-based MDR-TB DOT program is a substantially better option for me than getting my drugs at a health facility every day | 0 | 0 | 0 | 0 | 0 | 46 (100.0) | 46 |
| The community-based MDR-TB DOT program is more likely to result in my taking my medicines every day compared to getting my drugs at a health | 0 | 0 | 0 | 1 (2.2) | 0 | 45 (97.8) | 46 |
| The community-based MDR-TB DOT program is more likely to result in my being cured of my MDR TB compared to getting my drugs at ta health facility every | 0 | 0 | 0 | 1 (2.3) | 0 | 43 (97.7) | 44 |
| The community-based MDR-TB DOT program is a good fit for my health facility's needs | 0 | 0 | 0 | 4 (8.7) | 0 | 42 (91.3) | 46 |
| The community-based MDR-TB DOT program is consistent with my personal values | 0 | 0 | 0 | 8 (17.4) | 0 | 38 (82.6) | 46 |
| The community-based MDR-TB DOT program is consistent with my professional values | 0 | 0 | 0 | 11 (23.9) | 0 | 35 (76.1) | 46 |
| The community-based MDR-TB DOT program is a good fit for MDR TB patients' needs | 0 | 0 | 0 | 1 (2.2) | 0 | 44 (97.8) | 45 |
| The community-based MDR-TB DOT program works within my health facility's workflow | 0 | 1 (2.2) | 0 | 4 (8.7) | 0 | 41 (89.1) | 46 |
| I think it is a good idea for the community-based MDR-TB DOT program to be implemented | 0 | 0 | 0 | 3 (6.5) | 0 | 43 (93.5) | 46 |
| I trust the information the community-based MDR-TB DOT program provides to patients when they give them their drugs | 0 | 0 | 0 | 3 (6.5) | 0 | 43 (93.5) | 46 |

**Table S5: Acceptability and adaptation of community-based MDR-TB DOT program – providers’ perception**

| Items | Strongly Disagree | Somewhat disagree | Disagree | Somewhat agree | Agree | Strongly agree | Total  respondents |
| --- | --- | --- | --- | --- | --- | --- | --- |
| 1-1. I'm supportive of the community-based MDR-TB DOT program in the facility I work in | 0 | 1 (2.0) | 0 | 2 (3.9) | 0 | 48 (94.1) | 51 |
| 2-1. I don't think the community - based MDR-TB DOT program is good for MDR TB patients | 48 (94.1) | 0 | 0 | 0 | 0 | 3 (5.9) | 51 |
| 3-1. I'm enthusiastic about the community-based MDR-TB DOT program | 1 (2.0) | 0 | 0 | 3 (5.9) | 0 | 47 (92.1) | 51 |
| 4-1. I don't expect the community-based MDR-TB DOT program will result in any improvement for MDR TB patients | 44 (86.3) | 3 (5.9) | 0 | 0 | 0 | 4 (7.8) | 51 |
| 5-1. The community-based MDR-TB DOT program is very much needed by MDR TB patients | 0 | 0 | 0 | 0 | 0 | 51 (100.0) | 51 |
| 6-1. I don't know what the community-based MDR-TB DOT program is about | 47 (92.2) | 1 (1.9) | 0 | 0 | 0 | 3 (5.9) | 51 |
| 7-1. The community-based MDR-TB DOT program is substantially better option for MDR-TB patients than getting drugs at a health facility every day | 1 (1.9) | 0 | 0 | 3 (5.9) | 0 | 47 (92.2) | 51 |
| 8-1. The community-based MDR-TB DOT program will result in MDR-TB DOT patients taking their medicines everyday compared to getting their drugs at a health | 0 | 0 | 0 | 4 (7.8) | 0 | 47 (92.2) | 51 |
| 9-1. Having my patients receive their medicine through the community-based MDR-TB DOT program makes me feel more confident that they will be cured of MDR-TB | 0 | 0 | 0 | 1 (2.0) | 0 | 49 (98.0) | 50 |
| 10-1. It is easy for me to refer MDR-TB patients to the community-based MDR-TB DOT Program. | 1 (2.0) | 0 | 0 | 5 (9.8) | 0 | 45 (88.2) | 51 |
| 11-1. The community-based MDR-TB DOT program is less burdensome for me when treating MDR TB patients compared to our current standard of care | 0 | 3 (5.9) | 0 | 10 (19.6) | 0 | 38 (74.5) | 51 |
| 12-1. Referring patients to the community-based MDR-TB DOT program is complicated for my health facility | 29 (56.9) | 5 (9.8) | 0 | 9 (17.6) | 0 | 8 (15.7) | 51 |
| 13-1. The community-based MDR-TB DOT program does not integrate well with my health facility's workflow | 31 (60.8) | 7 (13.7) | 0 | 6 (11.8) | 0 | 7 (13.7) | 51 |
| 14-1. The community-based MDR-TB DOT program is good fit for my health facility's needs | 1 (2.0) | 1 (2.0) | 0 | 4 (7.8) | 0 | 45 (88.2) | 51 |
| 15-1. The community-based MDR-TB DOT-TB program is consistent with my personal values | 1 (2.0) | 0 | 0 | 8 (15.7) | 0 | 42 (82.3) | 51 |
| 16-1. The community-based MDR-TB DOT program is consistent with my professional values | 0 | 0 | 0 | 5 (9.8) | 0 | 46 (90.2) | 51 |
| 17-1. The community-based MDR DOT program is a good fit for MDR TB patients' needs. | 0 | 0 | 0 | 2 (3.9) | 0 | 49 (96.1) | 51 |
| 18-1. The community-based MDR-TB DOT program does not add to my workload | 5 (9.8) | 3 (5.9) | 0 | 10 (19.6) | 0 | 33 (64.7) | 51 |
| 19-1. I trust the information the community-based MDR-TB DOT program provides to patients when they give them their drugs | 0 | 0 | 0 | 0 | 0 | 50 (100.0) | 50 |
