# Supplementary material for: Community-based directly observed therapy is effective and results in better treatment outcomes for patients with multi-drug resistant tuberculosis in Uganda
Source: BMC Health Serv Res. 2023 Nov 13;23:1248. doi: 10.1186/s12913-023-10120-7 (PMC10644403; doi:10.1186/s12913-023-10120-7)
Supplement: Supplementary file 2 — Additional file 2. [file 12913_2023_10120_MOESM2_ESM.pdf]

TOOLS TO ASSSESS TREATMENT OUTCOMES OF A PILOT COMMUNITY BASED DOT MODEL FOR MDR TB IN UGANDA Version 2.1 September 07, 2021

A. Patient Interview Tool (to be administered at enrolment)	
1. Name of Research Assistant	
2. Health Facility (RRH/District):	
3. District	
4. Region	
5. Date of Interview	___ ___/___ ___ ___/___ ___ ___ __(DD/MMM/YYYY)
**Client Demographics**	
6. Patient TB No.:	
7. Treatment Initiation Health Facility	
8. Patients follow up for daily DOT	☐At follow up facility. Specify…………………………… ☐CBDOTs Program
9. Sex:	☐M ☐F
10. Date of Birth/ Age (completed years):	_......../…………/……….. ………………(years)__ ___ ___
11. Marriage Status	☐Single ☐ Married ☐ Divorced ☐ Separated ☐ widowed
12. Daily Income	☐ < $2 ☐ $2-5 ☐ $5-10 ☐ >$10
13. Occupation	
14. Education Level	☐Primary ☐ Secondary ☐ Tertiary
15. Area of Residence	(Village/LC Zone) ……………………………………. Parish/Subcounty ………………………………/…………………………………
**Medical History**	
16. Date initiated on MDR TB Treatment	___ ___/___ ___ ___/___ ___ ___ __(DD/MMM/YYYY)
17. Baseline culture	☐ Done ☐ Not Done
18. If done in no. 17 above, what is the result?	…………………………………….
19. In- patient at treatment Initiation?	☐ Yes ☐ No
20. Weight at treatment initiation date	
21. Height?	
22. BMI at treatment initiation?	
23. MUAC at treatment initiation?	
24. Duration on treatment (days)	
25. Current Treatment Modality	☐ DOT ☐ SAT
26. If on DOT (Type of DOT)	☐ Public facility ☐ Private facility ☐CHW/VHT
27. Any underlying co-morbidities	☐No ☐ yes
28. If yes in 27 above (Specify)	☐HIV ☐Diabetes ☐Hypertension ☐ Other (specify)……………………
29. Concomitant Medications being taken?	☐ Yes ☐ No
30. If yes in 29 above (Specify)	
31. MDR treatment Regimen (STR)/mSTR?	☐ Yes ☐ No
32. Community Model onto which the patient is enrolled onto (To be updated at a later date after the preferences study has been completed)	…………………………………..

B. Patient follow up log for recording daily community DOT, DOT provider and place in the community (to be updated at follow up visits)

Month/ Year: …………………………..

Calculate % adherence (total doses taken/total to have been taken) at the end of each month. (**Goal is 100%** and not to fall below 80%)

	Day of Month																															Total missed	%
	**1**	**2**	**3**	**4**	**5**	**6**	**7**	**8**	**9**	**10**	**11**	**12**	**13**	**14**	**15**	**16**	**17**	**18**	**19**	**20**	**21**	**22**	**23**	**24**	**25**	**26**	**27**	**28**	**29**	**30**	**31**		
DOT																																	
DOT Provider																																	
Place																																	
Patient concerns (Y/N)																																	

DOT

X = Directly observed

I = Incomplete dose (write an "I" in red pen)

✓ = Observed by treatment supporter

N = Not supervised

∅ = Drugs not taken (date and specify reason) in notes section below

If split doses are used, mark the upper left half for the morning dose and the lower right for the evening dose

DOT Provider

1= Family Member

2= Community Health worker

3= Expert Client point

4= Self-administered

5= Health workers

6= VHT

Place

W= work

H= Home

C= Community

HF= Health facility

Daily follow up notes in a given month:

**Date:**	**Key findings or concerns by the patient including any adverse drug reactions**	**Action taken**

Record of monthly treatment status and Culture

	Month 0 baseline	Month 1	Month 2	Month 3	Month 4	Month 5	Month 6	Month 7	Month 8	Month 9	Month 10	Month 11	Month 12
Treatment status													
Culture													
Smear													

Record Treatment status results

1 = Active treatment

2 = Interrupted treatment (Indicate number of days of interruption ……………….)

3= Lost to follow up

4= Died

Culture: Record as 1=Positive 2=Negative 3=contaminated 4= not done

Sputum smear: Record as 1= Positive 2= Negative 3= Note done

Documented Final treatment outcome: …………………………………

1= Cured

2= Treatment Completed

3= Treatment Failure

4= Loss to Follow up

5= Died

Nutritional Status at Discharge/treatment completion

MAUC= ………………….

Weight= …………………….

BMI= ……………………

Record of monthly treatment status and Culture

	Month 13	Month 14	Month 15	Month 16	Month 17	Month 18	Month 19	Month 20	Month 21	Month 22	Month 23	Month 24
Treatment status												
Culture												
Smear												

Record Treatment status results

1 = Active treatment

2 = Interrupted treatment (Indicate number of days of interruption ……………….)

3= Lost to follow up

4= Died

Culture: Record as 1=Positive 2=Negative 3=contaminated 4= not done

Sputum smear: Record as 1= Positive 2= Negative 3= Note done

Documented Final treatment outcome: …………………………………

1= Cured

2= Treatment Completed

3= Treatment Failure

4= Loss to Follow up

5= Died

Nutritional Status at Discharge/treatment completion

MAUC= ………………….

Weight= …………………….

BMI= ……………………

<u>Locator information (at enrolment and updated as need arises)</u>

Map of residence

Chairman of the village/LC zone

Name: …………………………………………

Tel. contact: ………………….
