# Supplementary material for: Community-based directly observed therapy is effective and results in better treatment outcomes for patients with multi-drug resistant tuberculosis in Uganda
Source: BMC Health Serv Res. 2023 Nov 13;23:1248. doi: 10.1186/s12913-023-10120-7 (PMC10644403; doi:10.1186/s12913-023-10120-7)
Supplement: Supplementary file 3 — Additional file 3. [file 12913_2023_10120_MOESM3_ESM.pdf]

Acceptability and adoption of the community-based MDR DOT will be assessed with the attached tool. The tool was adapted from an existing tool[1] and uses the Diffusion of Innovation Theory to identify dimensions of acceptability[2]. DOI can be used to examine how new interventions, such as the community-based MDR DOT intervention are adopted, both by organizations and by clients. Five characteristics of the innovation are outlined in the theory: relative advantage, compatibility, complexity, trialability, and observability.

Relative advantage represents the degree to which the innovation is perceived as better than the current standard of care. Compatibility represents the degree to which the innovation is compatible with values, experiences and needs of the adopters. Complexity represents how difficult the innovation is to implement. Trialability has to do with the degree to which the innovation can be tried and subsequently modified, while observability represents the degree to which the adoption of the innovation can be seen by others.

Both patients and providers will be interviewed to assess their acceptability and adoption of the community-based MDR DOT pilot. The patient tool will assess relative advantage and compatibility, while the provider tool will assess relative advantage, compatibility, and complexity. Trialability and observability were not relevant for this assessment. Both tools will also assess respondent's awareness, support, and enthusiasm for the community-based MDR TB program.

---

[1] Karla D. Wagner, Roy F. Oman, Krysti P. Smith, Robert W. Harding, Ashley D. Dawkins, Minggen Lu, Stephanie Woodard, Michelle N. Berry, Nancy A. Roget, "Another tool for the tool box? I'll take it!": Feasibility and acceptability of mobile recovery outreach teams (MROT) for opioid overdose patients in the emergency room, Journal of Substance Abuse Treatment, 2019. In press, https://doi.org/10.1016/j.jsat.2019.04.011.
[2] Rogers, E. M. (1995). Diffusion of innovations (4th ed.). New York: Free Press.

A) This section is to be administered to the patient

A. Patient Interview Tool (to be administered at the end of the study)	
1. Name of Research Assistant	
2. Health Facility (RRH/District):	
3. District	
4. Region	
5. Date of Interview	___ ___/___ ___ ___/___ ___ ___ __(DD/MMM/YYYY)
**Patient identification**	
6. Patient MDRTB clinic No.:	
7. Patients CBDOTs study number	

	**Patient tool**	
Awareness, support, and enthusiasm		
1	I'm supportive of the community-based MDR DOT program.	□ Strongly disagree<br>□ Somewhat disagree<br>□ Somewhat agree<br>□ Strongly agree
2	I don't think the community-based MDR DOT program is good for MDR TB patients.	□ Strongly disagree<br>□ Somewhat disagree<br>□ Somewhat agree<br>□ Strongly agree
3	I'm enthusiastic about the community-based MDR DOT program.	□ Strongly disagree<br>□ Somewhat disagree<br>□ Somewhat agree<br>□ Strongly agree
4	I don't expect the community-based MDR DOT program will result in any improvement for MDR TB patients like me.	□ Strongly disagree<br>□ Somewhat disagree<br>□ Somewhat agree<br>□ Strongly agree
5	The community-based MDR DOT program is very much needed by MDR TB patients like me.	□ Strongly disagree<br>□ Somewhat disagree<br>□ Somewhat agree<br>□ Strongly agree
6	I don't know what the community-based MDR DOT program is about.	□ Strongly disagree<br>□ Somewhat disagree<br>□ Somewhat agree<br>□ Strongly agree
Relative advantage:		
7	The community-based MDR DOT program is a substantially better option for me than getting my drugs at a health facility every day.	□ Strongly disagree<br>□ Somewhat disagree<br>□ Somewhat agree<br>□ Strongly agree
8	The community-based MDR DOT program is more likely to result in my taking my medicines everyday compared to getting my drugs at a health facility every day.	□ Strongly disagree<br>□ Somewhat disagree<br>□ Somewhat agree<br>□ Strongly agree
9	The community-based MDR DOT program is more likely to result in my being cured of my MDR TB compared to getting my drugs at a health facility every day.	□ Strongly disagree<br>□ Somewhat disagree<br>□ Somewhat agree<br>□ Strongly agree
Compatibility:		
10	The community-based MDR DOT program is a good fit for my health facility's needs.	□ Strongly disagree<br>□ Somewhat disagree<br>□ Somewhat agree<br>□ Strongly agree

11	The community-based MDR DOT program is consistent with my personal values.	□ Strongly disagree<br>□ Somewhat disagree<br>□ Somewhat agree<br>□ Strongly agree
12	The community-based MDR DOT program is consistent with my professional values	□ Strongly disagree<br>□ Somewhat disagree<br>□ Somewhat agree<br>□ Strongly agree
13	The community-based MDR DOT program is a good fit for MDR TB patients' needs.	□ Strongly disagree<br>□ Somewhat disagree<br>□ Somewhat agree<br>□ Strongly agree
14	The community-based MDR DOT program works within with my health facility's workflow.	□ Strongly disagree<br>□ Somewhat disagree<br>□ Somewhat agree<br>□ Strongly agree
15	I think it is a good idea for the community-based MDR DOT program to give.	□ Strongly disagree<br>□ Somewhat disagree<br>□ Somewhat agree<br>□ Strongly agree
16	I trust the information the community-based MDR DOT program provides to patients when they give them their drugs.	□ Strongly disagree<br>□ Somewhat disagree<br>□ Somewhat agree<br>□ Strongly agree

To be administered to the health provider (these include health care worker and community health workers)

**B. Health Provider Interview Tool (to be administered at the end of the study)**	
1. Name of Research Assistant	
2. Health Facility (RRH/District):	
3. District	
4. Region	☐
5. Date of Interview	___ ___/___ ___ ___/___ ___ ___ __(DD/MMM/YYYY)
**Health worker socio-demographics**	
6. Study number *(for CHW put previous study number)*	
7. Health Provider type	☐ Healthcare worker ☐Community health workers
8. If healthcare workers in 14 above, please specify	☐ Nurse ☐Clinical officer ☐ medical officer ☐other, specify………………
9. Date of Birth/ Age (completed years):	___ ___/___ ___ ___/___ ___ ___ __(DD/MMM/YYYY) (years)__ ___ ___
10. Sex:	☐M ☐F

	**Provider tool**	
Awareness, support, and enthusiasm		
1	I'm supportive of the community-based MDR DOT program in the facility I work in.	□ Strongly disagree<br>□ Somewhat disagree<br>□ Somewhat agree<br>□ Strongly agree
2	I don't think the community-based MDR DOT program is good for MDR TB patients.	□ Strongly disagree<br>□ Somewhat disagree<br>□ Somewhat agree<br>□ Strongly agree
3	I'm enthusiastic about the community-based MDR DOT program.	□ Strongly disagree<br>□ Somewhat disagree<br>□ Somewhat agree<br>□ Strongly agree
4	I don't expect the community-based MDR DOT program will result in any improvement for MDR TB patients.	□ Strongly disagree<br>□ Somewhat disagree<br>□ Somewhat agree<br>□ Strongly agree
5	The community-based MDR DOT program is very much needed by MDR TB patients.	□ Strongly disagree<br>□ Somewhat disagree<br>□ Somewhat agree<br>□ Strongly agree

6	I don't know what the community-based MDR DOT program is about.	□ Strongly disagree<br>□ Somewhat disagree<br>□ Somewhat agree<br>□ Strongly agree
Relative advantage:		
7	The community-based MDR DOT program is a substantially better option for patients than MDR TB patients than getting their drugs at a health facility every day.	□ Strongly disagree<br>□ Somewhat disagree<br>□ Somewhat agree<br>□ Strongly agree
8	The community-based MDR DOT program will result in MDR TB patients taking their medicines everyday compared to getting their drugs at a health facility every day.	□ Strongly disagree<br>□ Somewhat disagree<br>□ Somewhat agree<br>□ Strongly agree
9	Having my patients receive their medicine through the community-based MDR DOT program makes me feel more confident that they will be cured of MDR TB.	□ Strongly disagree<br>□ Somewhat disagree<br>□ Somewhat agree<br>□ Strongly agree
Complexity:		
10	It is easy for me to refer MDR TB patients to the community-based MDR DOT program.	□ Strongly disagree<br>□ Somewhat disagree<br>□ Somewhat agree<br>□ Strongly agree
11	The community-based MDR DOT program is less burdensome for me when treating MDR TB patients compared to our current standard of care.	□ Strongly disagree<br>□ Somewhat disagree<br>□ Somewhat agree<br>□ Strongly agree
12	Referring patients to the community-based MDR DOT program is complicated for my health facility.	□ Strongly disagree<br>□ Somewhat disagree<br>□ Somewhat agree<br>□ Strongly agree
13	The community-based MDR DOT program does not integrate well with my health facility's workflow.	□ Strongly disagree<br>□ Somewhat disagree<br>□ Somewhat agree<br>□ Strongly agree
Compatibility:		
14	The community-based MDR DOT program is a good fit for my health facility's needs.	□ Strongly disagree<br>□ Somewhat disagree<br>□ Somewhat agree<br>□ Strongly agree
15	The community-based MDR DOT program is consistent with my personal values.	□ Strongly disagree<br>□ Somewhat disagree<br>□ Somewhat agree<br>□ Strongly agree
16	The community-based MDR DOT program is consistent with my professional values	□ Strongly disagree<br>□ Somewhat disagree<br>□ Somewhat agree<br>□ Strongly agree

17	The community-based MDR DOT program is a good fit for MDR TB patients' needs.	□ Strongly disagree<br>□ Somewhat disagree<br>□ Somewhat agree<br>□ Strongly agree
18	The community-based MDR DOT program does not add to my workload.	□ Strongly disagree<br>□ Somewhat disagree<br>□ Somewhat agree<br>□ Strongly agree
19	I trust the information the community-based MDR DOT program provides to patients when they give them their drugs.	□ Strongly disagree<br>□ Somewhat disagree<br>□ Somewhat agree<br>□ Strongly agree
